# Association of a Combined Cancer Exhaustion Score with Circulating Tumor Cells and Outcome in Ovarian Cancer—A Study of the OVCAD Consortium

**DOI:** 10.3390/cancers13235865

**Published:** 2021-11-23

**Authors:** Eva Obermayr, Elena Ioana Braicu, Stephan Polterauer, Liselore Loverix, Nicole Concin, Linn Woelber, Sven Mahner, Jalid Sehouli, Toon Van Gorp, Ignace Vergote, Robert Zeillinger, Stefanie Aust

**Affiliations:** 1 Department of Obstetrics and Gynecology, Comprehensive Cancer Center-Gynecologic Cancer Unit, Medical University of Vienna, 1090 Vienna, Austria; eva.obermayr@muv.ac.at (E.O.); stephan.polterauer@meduniwien.ac.at (S.P.); stefanie.aust@meduniwien.ac.at (S.A.); 2 European Competence Center for Ovarian Cancer, Department of Gynecology, Berlin Institute of Health, Charité-Universitätsmedizin Berlin, Corporate Member of Freie Universität Berlin, Humboldt-Universität zu Berlin, 13353 Berlin, Germany; ioana@braicu.de (E.I.B.); Jalid.sehouli@charite.de (J.S.); 3 Division of Gynecological Oncology, Department of Obstetrics and Gynecology, Leuven Cancer Institute, University Hospitals Leuven, Katholieke Universiteit Leuven, 3000 Leuven, Belgium; liselore.loverix@uzleuven.be (L.L.); toon.vangorp@uzleuven.be (T.V.G.); ignace.vergote@uzleuven.be (I.V.); 4 Department of Obstetrics and Gynecology, Innsbruck Medical University, 6020 Innsbruck, Austria; nicole.concin@i-med.ac.at; 5 Department of Gynecology and Gynecologic Oncology, University Medical Center Hamburg-Eppendorf, 20246 Hamburg, Germany; linn_woelber@gmx.de (L.W.); Sven.Mahner@med.uni-muenchen.de (S.M.); 6 Department of Obstetrics and Gynecology, University Hospital, LMU Munich, 81377 Munich, Germany

**Keywords:** circulating tumor cells, epithelial ovarian cancer, inflammation, hypoalbuminemia, biomarker

## Abstract

**Simple Summary:**

Advances in circulating tumor cells (CTCs) research could improve the way we diagnose, treat and monitor epithelial ovarian cancer (EOC) patients. In this study, we investigated the interplay between inflammation-induced systemic catabolic markers and the presence of CTCs and patient outcome, and showed that the presence of CTCs goes hand-in-hand with an increased protein turnover and immune activation. We integrated the information provided by measuring the plasma levels of albumin, tryptophan and its metabolite kynurenine in a combined score for cancer exhaustion (CCES). Our study indicates that not only could the CCES be a useful prognostic biomarker in women with EOC, but also that the interaction between the systemic microenvironment and CTCs is worth investigating in further studies.

**Abstract:**

We investigated the prognostic role of systemic characteristics for cancer exhaustion and the presence of circulating tumor cells (CTCs) in primary epithelial ovarian cancer (EOC) patients. We included 185 patients in this multicenter study with a median follow-up time of 10.25 years. Albumin, c-reactive protein (CRP) and the kynurenine to tryptophan ratio (Kyn/Trp) as well as the CTC-related marker cyclophilin C (PPIC) were obtained before primary therapy and were correlated to the respective clinical and outcome data. The information provided by albumin and Kyn/Trp was integrated in a combined score for cancer exhaustion (CCES). A high CCES characterized by hypoalbuminemia and a high Kyn/Trp was associated with both decreased overall and progression-free survival, independent from other known prognostic factors in a multivariable analysis. The presence of PPIC-positive CTCs was significantly associated with a high CCES, highlighting that the interplay between the systemic microenvironment and CTCs should be considered in “liquid biopsy” biomarker assessment.

## 1. Introduction

Ovarian cancer is the 7th most common cancer in women (and the 18th most common cancer overall) worldwide [1]. This cancer has a low survival and is the eighth most common cause of cancer death in women [2]. 

The disease is generally advanced when it is diagnosed, and no adequate method has been established so far for advancing early diagnostic or screening. Within the past decade, the diagnostic, prognostic and therapeutic potential of circulating tumor cells (CTCs) has been demonstrated in several studies and solid tumors [3]. In addition, in epithelial ovarian cancer (EOC), CTCs emerge as a noninvasive tool, identifying patients at risk for early recurrence or death [4,5,6,7]. Still, comprehensive studies of the biological and clinical implications of CTCs are needed to advance them toward clinical use. 

We previously published data on the expression of the cyclophilin C gene (PPIC) as promising marker for CTCs in EOC [8]. We further described the kynurenine-to-tryptophan ratio (Kyn/Trp ratio) as a biomarker for immune activation and its association with the presence of CTCs, an aspect that still needs to be further developed in CTC research. Besides immunological conditions, inflammatory processes are presumably linked to the shedding of CTCs into the (lympho-) vascular system [9]. 

Studies of cancer risk and molecular carcinogenesis suggest a role for inflammation in cancer development progression and spread [10]. Additionally, markers for inflammation as well as scores, e.g., the Glasgow Prognostic Score, have a prognostic relevance in EOC as well as other solid tumors; however, they are not currently used in everyday clinical practice and in the management of EOC patients.

Proinflammatory cytokines such as IL1, IL6 and TNF and growth factors are released as part of the systemic inflammatory response to a tumor, affecting albumin synthesis as well as CRP secretion [11,12]. Malnutrition and an increased turnover of albumin by tumors adds up to lower albumin levels observed in cancer patients [13]. Hypoalbuminemia has been associated with worse prognosis in a variety of solid tumors, including ovarian cancer [14]. An increase in CRP concentration has been shown to be associated with poorer survival in cancer patients, not only in the palliative situation [15] but also with primary operable cancer [16].

The role of systemic inflammatory conditions related to cancer exhaustion and cancer dissemination, particularly the presence of CTCs in EOC patients, remains largely uncharacterized. Comprehensive studies of the biological and clinical implications of CTCs are still needed to advance them toward clinical use. Thus, in the present study, we determined whether specific systemic characteristics for cancer exhaustion correlate and are associated with the presence of circulating tumor cells and clinical outcome in patients with primary advanced EOC recruited within the multicentered OVCAD study [17].

## 2. Results

### 2.1. Patient Characteristics

From the 275 patients included in the OVCAD study, sufficient data to calculate the CCES were available in 185 patients (see Supplementary Figure S1). The median follow-up time was 10.25 years (range 1 month to 13.67 years). Disease recurrence was observed in 80.5% of the patients, and 71.4% died within the observation period. The detailed patient characteristics at baseline are shown in Table 1.

Albumin concentrations were measured in 143 patients, with a mean of 40.5 g/L (range 15.2–80.0 g/L; median 40.3 g/L). Hypoalbuminemia, according to the lower limit of normal (LLN) of 35.0 g/L, was observed in 30 (16.2%) patients. CRP was assessed in 150 patients with a mean of 3.92 mg/dL (range 0.09–40.06 mg/dL; median 1.99 mg/dL), with 50 patients (33.3%) having CRP levels below the prognostic threshold previously set at 1.0 mg/dL [18]. The ratio of kynurenine to tryptophan (Kyn/Trp) had been assessed in 177 patients with a mean ratio of 71.2 (range 20.7–348.6; median 57.3). In 108 (58.4%) patients, a high Kyn/Trp ratio beyond the prognostic cut-point set at 52.5 according to our earlier study [19] was observed.

### 2.2. Combined Cancer Exhaustion Score (CCES)

In a next step, we pooled the information provided by albumin and Kyn/Trp, both being associated with cancer exhaustion, to a combined score for cancer exhaustion. The 64 patients with albumin > LLN and concomitant low Kyn/Trp were assigned to the CCES 0 group. In contrast, 121 patients were assigned to the CCES 1 group, due to either hypoalbuminemia (*n* = 30) or a high Kyn/Trp ratio (*n* = 91).

The association of CCES with clinicopathological characteristics of the patients is shown in Table 2. Hypoalbuminemia or a high Kyn/Trp ratio (CCES 1) was significantly associated with advanced stage of the disease at diagnosis, an ECOG (Eastern Cooperative Oncology Group) performance status of ≥1, presence of ascitic fluid, and high CRP plasma levels. In contrast, we did not observe any association of CCES with patient age, histological type of the tumor, type of primary treatment and residual disease after surgery. 

### 2.3. Association of CTC-Related Markers with Biochemical Parameters

We compared the plasma levels of albumin and CRP in patients who had been assigned as CTC-positive and CTC-negative due to the presence or absence of the CTC-related marker cyclophilin C (PPIC) in the mononuclear cell fraction of the blood samples [8]. PPIC was assessed in 173 of the 185 patients using qPCR, resulting in 34 (19.6%) PPIC-positive and 139 (80.3%) PPIC-negative patients (see Supplementary Figure S1). The presence of PPIC-positive CTCs was significantly associated with higher CRP levels and a higher Kyn/Trp ratio (Table 3). Thus, CTC-positive patients were significantly more likely to have a high CCES than CTC-negative patients (85.3% vs. 60.4%, chi-square *p* = 0.006).

### 2.4. Association with Outcome

The impact of albumin, CRP and the CCES on outcome was assessed using Kaplan–Meier analysis. Hypoalbuminemia was significantly associated with worse overall survival (median 38 vs. 57 months; log-rank *p* = 0.009), but not with shorter PFS. CRP levels >1 mg/dL did not have a prognostic impact on OS nor on PFS. Patients with a high CCES had a significantly worse OS (45 vs. 70 months; log-rank *p* < 0.001) and PFS (18 vs. 24 months; log-rank *p* = 0.011).

In multivariate Cox regression analysis including patient age, FIGO stage, and residual tumor load, and adjusting the hazard for the histological type of the tumor (HGSOC vs. other), a high CCES was independently associated with both decreased overall and progression-free survival (Table 4). 

**Figure 1:**
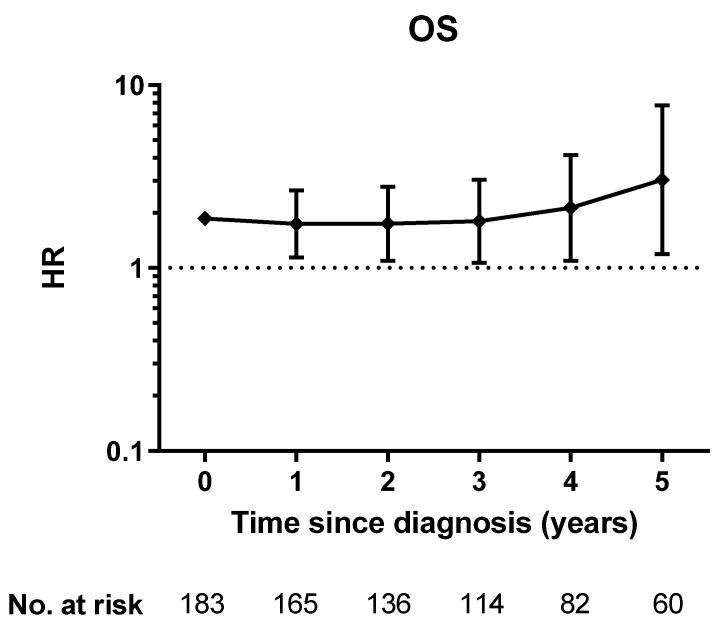
Plot of adjusted hazard ratio (diamonds) of CCES 1 vs. CCES 0 in patients who had already survived up to 5 years after diagnosis, 95% CIs are depicted as bars.

In order to find out whether the independent prognostic impact of CCES on survival was also relevant in patients who had already survived a number of years, we performed a conditional survival analysis. The hazard of death was calculated in a multiple Cox regression model using the same covariates as above (age, FIGO stage, residual tumor load, CCES). As a result, a high CCES was also an independent risk factor for death in the subgroup of patients who had already survived for up to 5 years after diagnosis (Figure 1). 

## 3. Discussion

In the present study, specific systemic characteristics for cancer exhaustion were correlated with clinical outcome data and the presence of PPIC-positive circulating tumor cells in EOC patients. The majority of patients (65%) had a high combined cancer exhaustion score (CCES), due to hypoalbuminemia or a high kynurenine to tryptophan (Kyn/Trp) ratio. A significant association between a high CCES and advanced tumor stage, the presence of ascites of more than 500 mL, and a worse ECOG performance status was observed. Additionally, patients with a high CCES had significantly higher CRP levels. 

We previously described the Kyn/Trp ratio as a biomarker for immune activation in EOC [19]. By combining hypoalbuminemia and an increased Kyn/Trp ratio in the CCES, our aim was to further investigate the impact of inflammation-induced systemic catabolic markers on outcome as well as the presence of PPIC positive circulating tumor cells. In our study, a high CCES was an independent prognostic factor for worse PFS and OS. In multiple analysis, the impact on OS of (i) the presence of residual tumor after debulking surgery and (ii) an increased CCES was comparable (HR 1.721 and HR 1.867, respectively). A high baseline CCES was significantly associated with an increased risk for death not only in the entire cohort of patients, but even in those patients who had already survived for up to 5 years. In our previous study, the presence of PPIC mRNA in the enriched blood samples was associated with worse survival; however, the prognostic impact of PPIC-positive CTCs was only observed after completion of the adjuvant treatment, and not at baseline [8]. For this reason, PPIC-positive CTCs at baseline were not prognostic in the present study as well.

Albumin comprises about 55% of the total serum protein, and cancer-associated hypoalbuminemia is associated with a variety of systemic changes in response to the tumor. Hypoalbuminemia is indicative of an increased catabolism related to an inflammatory systemic response, suppression of albumin synthesis and an increased vascular permeability followed by a shift of albumin from the intravascular sector towards the interstitium [20]. Low albumin was shown to be prognostic in many cancer types [14,21,22]. Our study confirms that EOC patients with hypoalbuminemia, diagnosed shortly before debulking surgery, had a significantly worse OS. 

Another systemic change observed in cancer patients is an altered tryptophan metabolism, primarily mediated by increased tryptophan 2,3-dioxygenase (TDO) and indoleamine 2,3-dioxygenase 1 (IDO1) activities [23]. IDO is activated by proinflammatory cytokines of the T helper 1 (Th1) type immune response, particularly interferon gamma (IFN-γ), and is reflected by higher levels of the tryptophan metabolite kynurenine. An elevated ratio of kynurenine to tryptophan (Kyn/Trp) as a measure of IDO activation was observed in numerous cancer types (reviewed by [24]). In addition to increased kynurenine levels, hypoalbuminemia can also cause a high Kyn/Trp ratio, because less albumin will be available to bind tryptophan, and consequently the plasma concentration of free tryptophan will increase. As IDO is induced under similar conditions as hypoalbuminemia, the Kyn/Trp ratio is likely to be influenced by both as a function of proinflammatory cytokines released in the circulation [25]. The fact that the presence of concomitant systemic diseases that might affect the immune system and the proinflammatory markers is not documented in the patient history is certainly a limitation of our study. 

To better understand cancer dissemination and progression, not only the interplay of tumor cells and their local primary microenvironment, but also the interplay between the systemic microenvironment and CTCs needs to be considered. We observed that 85% of patients with PPIC-positive CTCs had an increased CCES. In this regard, the expression of IDO may also play an important role in evading immune cell attack. In EOC, Inaba et al. showed that high IDO expression in the tumor cells correlated with a reduced number of CD8+ tumor-infiltrating lymphocytes (TILs) and that administration of an IDO inhibitor decreased the tumor peritoneal dissemination [26]. Furthermore, accumulation of kynurenine promotes the differentiation of CD4+ T cells into immunosuppressive regulatory (Treg) cells [27,28]. 

We could also observe that CTCs were associated with increased CRP levels. CRP has been reported to be both, prognostic of outcome and predictive of response to chemotherapy, in a range of cancers [29]. In patients with EOC, CRP was shown to be highly expressed and to be an independent prognostic variable [18]. During the acute phase of inflammation, interleukin-6 is the principal inducer of CRP gene expression. The association of CRP and CTCs was recently evaluated in a renal cell carcinoma study, further demonstrating a strong correlation of both parameters with coagulation [30]. More specifically, the authors suggest the formation of neutrophil extracellular traps being the missing link between inflammation and coagulation, which in turn may protect CTCs in the hostile environment. 

Our study highlights the importance of a combined score for cancer exhaustion. Both albumin levels and the formation of kynurenine are influenced by inflammation, and the deviation of either parameter from normal levels indicated a higher risk to die from the disease, even in those patients who had already survived for a rather long time. The measurement of albumin is sensitive, specific and reproducible at low cost. Our results suggest that implementing the measurement of tryptophan metabolites in the clinical routine could be useful for identifying high-risk patients and for establishing personalized treatment options.

## 4. Materials and Methods

### 4.1. Study Design

Patients with primary EOC were recruited from 2005 to 2008 within the multicentered OVCAD study, a 6th Framework Program Project (LSHC-CT-2005-018698) of the European Union. The 275 included patients were diagnosed at advanced stage (FIGO stage IIB-IV, according to the International Federation of Gynecology and Obstetrics), and all received standard treatment consisting of debulking surgery and platinum-based combination chemotherapy. Response to treatment was evaluated by experienced gynecological oncologists of the participating university centers according to the WHO criteria, i.e., by an increase in the nadir serum CA-125 level according to the GCIG criteria and by radiological confirmation [31]. Detailed inclusion and exclusion criteria, together with clinical data, have already been presented in previous studies published by the OVCAD group [17]. Written informed consent was obtained from all patients. The study protocol was approved by the local ethics committees of the participating OVCAD partners (EK207/2003, ML2524, HEK190504, EK366, and EK260).

### 4.2. Blood Samples and CTC Analyses

Blood samples were drawn prior to primary surgery or neo-adjuvant chemotherapy if applied. All samples were processed on the same day, using a two-layer density gradient centrifugation as described previously to obtain a plasma fraction and a monocyte blood fraction possibly containing CTCs. In the monocyte fraction, the expression levels of 11 markers—among them the cyclophilin C (PPIC) encoding gene—were analyzed with qPCR [8].

### 4.3. Biochemical Analyses

In the blood plasma fraction, albumin was assayed with bromocresol green using routine clinical chemical photometric analyzers. Hypoalbuminemia was defined as an albumin concentration less than the lower limit of normal (LLN) of 35 g/L [32]. CRP was measured by a modified latex-enhanced immuno-turbidimetric assay using a routine clinical CRP Latex kit according to the manufacturer’s instructions. Based on previous studies using 1 mg/dL as prognostic cut-off level for CRP in EOC [18], we defined levels ≤1 mg/dL as normal in this study. Tryptophan and kynurenine had been quantified previously using HPLC on reversed phase as described, with a ratio of kynurenine to tryptophan (Kyn/Trp) above the median of 52.5 being independently associated with poor prognosis [19]. Correspondingly, that ratio was used as prognostic cut-off in this study. 

### 4.4. Combined Cancer Exhaustion Score (CCES)

A combined score for cancer exhaustion (CCES) was introduced taking albumin levels and Kyn/Trp into account. The CCES was defined as follows: score 1 indicated hypoalbuminemia or a Kyn/Trp ratio ≥ 52.5, and score 0 normal albumin levels and a Kyn/Trp ratio < 52.5, concomitantly.

### 4.5. Statistics

The associations of the CCES with clinico-pathological characteristics were assessed using the Pearson chi-square and Fisher’s exact test where appropriate. A Bonferroni correction was applied to correct for multiple testing. Differences in albumin between CTC positive and negative cases as determined by the expression of the cyclophilin C gene (PPIC) were calculated using the two-sided *t*-test, whereas differences in CRP, CA-125, and Kyn/Trp were assessed using the Mann–Whitney-*U*-test.

Clinical endpoints were progression-free survival (PFS) and overall survival (OS). Both endpoints were defined as the time between first diagnosis and recurrence or death due to any cause, respectively. Kaplan–Meier survival analyses and log-rank testing were used to compare survival outcomes of the patient stratified by albumin (hypoalbuminemia vs. normal), CRP (≤1 mg/dL vs. >1 mg/dL) and the CCES (high vs. low). The Cox proportional hazards regression was used to determine univariate and multiple hazards ratios for PFS and OS stratified by histological type/grade of the primary tumor into high-grade serous ovarian cancer (HGSOC) and low-grade serous ovarian cancer (LGSOC) or other histological types. Covariates were patient age (≥55 years vs. <55 years), FIGO stage (IIIC and IV vs. IIA-IIIB), performance status of the patients according to the Eastern Cooperative Oncology Group (ECOG performance score 1–2 vs. 0), residual tumor load after surgery (yes vs. no), CCES, (1, high vs. 0, low), CTCs (PPIC-positive vs. PPIC-negative), CRP (>1 mg/dL vs. <1 mg/dL), albumin (hypoalbuminemia vs. >LLN of 35 g/L), Kyn/Trp (high ≥ 52.5 vs. low < 52.5).

Conditional overall survival probabilities were calculated in a multiple Cox regression model using the same covariates for patients who had already survived for 1, 2, 3, 4 and 5 years after diagnosis. 

Statistics were performed using SPSS v21.0 (SPSS Inc., Chicago, IL, USA; RRID: SCR_002865) and GraphPad Prism v9.1.2. The level of significance was set at *p* < 0.05.

## 5. Conclusions

In conclusion, this study suggests that CTC-positive patients had significantly more often a high CCES compared to CTC-negative patients. Additionally, a high combined cancer exhaustion score might be a useful prognostic biomarker in women with EOC. To improve the way we treat EOC, the advances in CTC research need to be integrated in the design of well-controlled trials.

## 6. Patents

E.O. and R.Z. filed a patent application for using PPIC as a novel tumor marker in ovarian cancer.

## Figures and Tables

**Table 1:** Baseline characteristic of the patients. LGSOC, low grade serous ovarian cancer; HGSOC, high grade serous ovarian cancer; ^a^ clear cell (*n* = 2), endometrioid (*n* = 7), mixed epithelial (*n* = 7), undifferentiated (*n* = 8); ^b^ plasma albumin ≤ 35.0 g/L. NA not assessed.

	*n* (%)
All	185
Age	
Median, range (years)	59 (18–83)
<55 years	71 (38.4%)
≥55 years	114 (61.6%)
FIGO stage	
IIA-IIIB	18 (9.7%)
IIIC	135 (73.0%)
IV	32 (17.3%)
ECOG performance status	
0	96 (51.9%)
1	70 (37.8%)
2	10 (5.4%)
NA	9 (4.9%)
Histotype	
LGSOC	16
HGSOC	145
other ^a^	24
Primary treatment	
Neoadjuvant chemotherapy	34 (18.4%)
Primary debulking surgery	151 (81.6%)
Residual tumor after surgery	
0 cm	123 (66.4%)
>0 cm	61 (33.0%)
NA	1 (0.1%)
Ascites	
<500 mL	50 (27.0%)
≥500 mL	100 (54.1%)
NA	35 (18.9%)
Albumin	
Median, range (g/L)	40.3 (15.2–80.0)
hypoalbuminemia ^b^	30 (16.2%)
normal levels	113 (61.1%)
NA	72 (38.9%)
Kyn/Trp	
Median, range	57.3 (20.7–348.6)
<52.5	69 (37.3%)
≥52.5	108 (58.4%)
NA	8 (4.3%)
CRP	
Median, range (mg/dL)	1.99 (0.09–40.06)
<1 mg/dL	34 (18.4%)
≥1 mg/dL	139 (75.1%)
NA	12 (6.5%)
CTCs	
PPIC-positive	34 (18.4%)
PPIC-negative	139 (75.1%)
NA	12 (6.5%)

**Table 2:** Association of the combined cancer exhaustion score (CCES) with patients’ characteristics. Patients with hypoalbuminemia or a high Kyn/Trp ratio were assigned to the CCES 1 group, and those with normal albumin and low Kyn/Trp to the CCES 0 group. Pearson’s chi-square was used to assess the association of each parameter and CCES. LGSOC, low grade serous ovarian cancer; HGSOC, high grade serous ovarian cancer; ^a^ clear cell (*n* = 2), endometrioid (*n* = 7), mixed epithelial (*n* = 7), undifferentiated (*n* = 8). NA not assessed. * Bonferroni corrected *p*-value.

	Total	CCES 1 *n* (%)	CCES 0 *n* (%)	*p*	*p* *
	185	121 (65.4)	64 (34.6)		
FIGO stage				0.039	n.s.
IIA-IIIB	18	10 (8.3)	8 (12.5)		
IIIC	135	84 (69.4)	51 (79.7)		
IV	32	27 (22.3)	5 (7.8)		
ECOG performance status				0.010	0.041
0	96	55 (45.5)	41 (64.1)		
1–2	80	61 (50.4)	19 (29.7)		
NA	9	5 (4.1)	4 (6.3)		
Histotype				0.265	n.s.
LGSOC	16	8	8		
HGSOC	145	95	50		
other ^a^	24	18	6		
Ascites				0.001	0.004
<500 mL	42	18 (14.9)	24 (37.5)		
≥500 mL	136	97 (80.2)	39 (60.9)		
NA	7	6 (5.0)	1 (1.6)		
CRP				<0.001	<0.001
<1 mg/dL	50	18 (14.9)	32 (50.0)		
≥1 mg/dL	100	82 (67.8)	18 (28.0)		
NA	35	21 (17.4)	14 (21.9)		

**Table 3:** Association of CTCs and albumin, CRP, CA-125 and the ratio of kynurenine to tryptophan levels in the blood plasma. The two-sided *t*-test (*) and the Mann–Whitney *U*-test were used to assess the association of each parameter and the presence of CTCs indicated by overexpression of the PPIC gene in the mononuclear blood cell fraction.

	CTC-Positive *n* = 34	CTC-Negative *n* = 139	*p*
Albumin (g/L)			0.149 *
median	37.0	41.9	
IQR	33.0–40.7	38.0–45.6	
Kyn/Trp			<0.001
median	76.6	54.7	
IQR	60.7–94.5	40.4–73.4	
CRP (mg/dL)			0.001
median	4.33	1.52	
IQR	1.46–7.51	0.50–4.50	
CA-125 (U/mL)			0.054
Median	705.9	426.2	
IQR	293.8–1411.6	105.7–1103.4	

**Table 4:** Cox’s proportional hazard regression for overall and progression-free survival. CI, confidence interval; HR, hazard ratio adjusted for histological type (HGSOC high grade serous ovarian cancer vs. LGSOC low grade serous ovarian cancer and other types); * not included in the final multiple regression model.

	Univariate			Multiple		
	HR	95% CI	*p*	HR	95% CI	*p*
Overall survival						
Age						
<55 years	1			1		
≥55 years	1.921	1.317–2.801	0.001	1.669	1.139–2.445	0.009
FIGO stage						
IIA-IIIB	1			1		
IIIC	2.468	1.132–5.382	0.023	2.231	1.015–4.906	0.046
IV	5.089	2.165–11.962	<0.001	3.544	1.489–8.437	0.004
ECOG performance status				*		
0	1					
1	1.199	0.840–1.712	0.317			
Residual tumor load						
no	1			1		
yes	2.165	1.508–3.107	<0.001	1.721	1.193–2.482	0.004
CCES						
0	1			1		
1	2.234	1.503–3.320	<0.001	1.867	1.247–2.796	0.002
CTCs				*		
PPIC-negative	1	1				
PPIC-positive	1.220	0.786–1.896	0.376			
CRP				*		
<1 mg/dL	1					
>1 mg/dL	1.137	0.754–1.716	0.540			
Albumin				*		
>35 g/L	1					
≤35 g/L	1.830	1.143–2.930	0.012			
Kyn/Trp				*		
<median	1					
≥median	2.133	1.449–3.140	<0.001			
Progression-free survival						
Age						
<55 years	1			1		
≥55 years	1.648	1.154–2.353	0.006	1.431	0.995–2.059	0.053
FIGO stage						
IIA-IIIB	1	1		1		
IIIC	1.803	0.956–3.400	0.069	1.614	0.845–3.081	0.147
IV	3.319	1.604–6.869	0.001	2.098	0.985–4.468	0.055
ECOG performance status				*		
0	1					
1	1.177	0.833–1.663	0.355			
Residual tumor load						
no	1			1		
yes	2.598	1.811–3.727	<0.001	2.228	1.531–3.241	<0.001
CCES						
0	1			1		
1	1.747	1.222–2.496	0.002	1.571	1.080–2.286	0.018
CTCs				*		
PPIC-negative	1					
PPIC-positive	1.191	0.780–1.818	0.419			
CRP				*		
<1 mg/dL	1					
>1 mg/dL	1.369	0.921–2.034	0.120			
Albumin				*		
>35 g/L	1					
≤35 g/L	1.253	0.785–2.002	0.344			
Kyn/Trp				*		
<median	1					
≥median	1.752	1.231–2.494	0.002			

## Data Availability

The data presented in this study are available on request from the corresponding author.

## Supplementary Materials

The following are available online at https://www.mdpi.com/article/10.3390/cancers13235865/s1, Supplementary Figure S1: Case flow diagram of the included OVCAD study patients and samples.
