# Supplementary figures and images for: Association of a Combined Cancer Exhaustion Score with Circulating Tumor Cells and Outcome in Ovarian Cancer—A Study of the OVCAD Consortium
Source: Cancers (Basel). 2021 Nov 23;13(23):5865. doi: 10.3390/cancers13235865 (PMC8657288; doi:10.3390/cancers13235865)

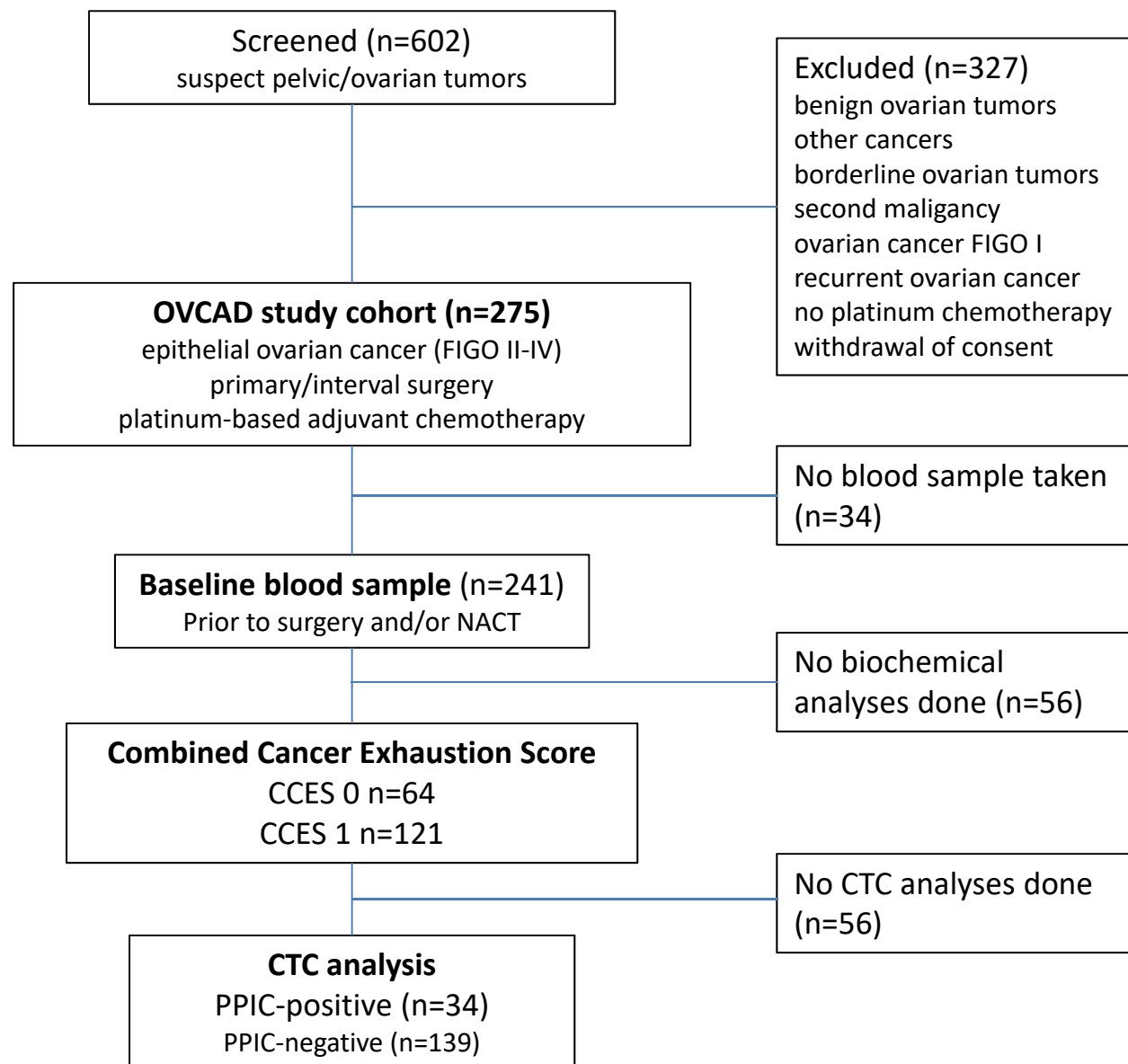

**Supplementary Figure S1.** Case flow diagram of the included OVCAD study patients and samples.

Supplement: Supplementary file 1 [file cancers-13-05865-s001.zip › cancers-1422183-supplementary.pdf]
